# Psychometric evaluation of the canine brief pain inventory in a Swedish sample of dogs with pain related to osteoarthritis

**DOI:** 10.1186/s13028-017-0311-2

**Published:** 2017-07-01

**Authors:** Ann Essner Essner, Lena Zetterberg, Karin Hellström, Pia Gustås, Hans Högberg, Rita Sjöström

**Affiliations:** 1 Department of Neuroscience, Faculty of Physiotherapy, Uppsala University, Husargatan, Biomedical Centre, Box 593, 751 24 Uppsala, Sweden; 2 Evidensia Djurkliniken Gefle, Norra Gatan 1, 803 21 Gävle, Sweden; 3 Department of Clinical Sciences, Faculty of Veterinary Medicine and Animal Husbandry, Swedish University of Agricultural Sciences, Box 7054, 750 07 Uppsala, Sweden; 4 Department of Health and Caring Sciences, Faculty of Health and Occupational Studies, University of Gävle, 801 76 Gävle, Sweden; 5 Unit of Research Education and Development, Region Jämtland Härjedalen, Box 654, 831 27 Östersund, Sweden; 6 Department of Community Medicine and Rehabilitation, Faculty of Physiotherapy, Umeå University, 901 87 Umeå, Sweden

**Keywords:** CBPI, Dogs, Measurement properties, Osteoarthritis, Pain, Physiotherapy, Rehabilitation

## Abstract

**Background:**

To evaluate intervention, implement evidence-based practice and enhance the welfare of dogs with naturally occurring osteoarthritis (OA), access to valid, reliable and clinically relevant outcome measures is crucial for researchers, veterinarians and rehabilitation practitioners. The objectives of the present study were to translate and evaluate psychometric properties, in terms of internal consistency and construct validity, of the owner-reported measure canine brief pain inventory (CBPI) in a Swedish sample of dogs with pain related to OA.

**Results:**

Twenty-one owners of clinically sound dogs and 58 owners of dogs with pain related to OA were included in this observational and cross-sectional study. After being translated according to the guidelines for patient-reported outcome measures, the CBPI was completed by the canine owners. Construct validity was assessed by confirmatory factor analysis, by repeating the principal component analysis and by assessing for differences between clinically sound dogs and dogs with pain related to OA. Internal consistency was estimated by Cronbach’s α. Confirmatory factor analysis was not able to confirm the factor-structure models tested in our sample. Principal component analysis showed a two-component structure, pain severity and pain interference of function. Two components accounted for 76.8% of the total variance, suggesting an acceptable fit of a two-component structure. The ratings from the clinically sound dogs differed from OA dogs and showed significantly lower CBPI total sum. Cronbach’s α was 0.94 for the total CBPI, 0.91 for the pain severity and 0.91 for the pain interference of function.

**Conclusions:**

The results indicate that the translated version of the CBPI is valid for use in the Swedish language. The findings suggest satisfying psychometric properties in terms of high internal consistencies and ability to discriminate clinically sound dogs from OA dogs. However, based on the confirmatory factor analysis, the original factor structure in the CBPI is not ideally suited to measure pain related to OA in our sample and the hypothesis of the presented two-factor structure was rejected. Further research needs to be conducted to determine whether the original psychometric results from CBPI can be replicated across different target groups and particularly with larger sample size.

**Electronic supplementary material:**

The online version of this article (doi:10.1186/s13028-017-0311-2) contains supplementary material, which is available to authorized users.

## Background

To evaluate intervention, implement evidence-based practice and enhance the welfare of dogs with naturally occurring osteoarthritis (OA), access to valid, reliable and clinically relevant outcome measures is crucial for researchers, veterinarians and rehabilitation practitioners [1–3]. In human medicine, the use of patient-reported outcome measures in clinical practice is essential in patient-centered healthcare to follow the impact of interventions, choose the appropriate treatment and to capture the effect perceived by the patient [4]. Brief pain inventory is a well-known self-reported generic measure that originally was developed to capture pain intensity and pain interference with function in daily life in human cancer patients [5]. Over the years the human brief pain inventory has been validated across languages and cultures and in different diseases [6–8]. Brown et al. [9, 10] adapted the instrument for use in dogs with OA related pain and pain in bone cancer. The canine brief pain inventory (CBPI) is a questionnaire intended to capture different dimensions of dog owners’ perceptions of canine OA pain. To measure pain severity and how pain interferes with function in the daily life in dogs, the answers of the items in the questionnaire are given by a person living in the same household as the dog of interest, i.e. the owner of the dog [9]. Other owner-reported instruments have been used such as the Helsinki Chronic Pain Index [11], the Liverpool osteoarthritis in dogs [12] and the canine orthopedic index [13], besides the CBPI, in canine OA. Pain is a subjective unpleasant sensory and emotional experience in animals, as well as in humans, and their inability to communicate their experience in words makes it impossible to use self-reporting instruments to directly assess pain severity and pain interference with function [1]. Recognizing and assessing pain in a dog is challenging and even in humans, who are able to communicate their pain experience, it has been shown that there may be discrepancies in the agreement between the perceived intensity of pain assessed by the individual experiencing pain and the individual’s pain as estimated by a parent [14]. Owner-reported pain instruments are based on canine behavioural changes affected by pain and the ability of the naïve observers, i.e. the owners, to recognize the behavioural signs in their dogs [15]. It has been shown by Hielm-Björkman et al. [16] that the ability of untrained owners to report the level of pain associated with OA on a visual analogue scale is limited, as they may not recognize the subtle signs of pain. Pain associated with OA may be manifested as changes in movement behaviour in the dog and gait evaluation during pain management is widely used in clinical settings. However, visual movement assessment and assigning levels and grades of lameness have shown poor intra- and inter-rater reliability among owners [17] and veterinarians [17, 18]. Hence, there is a challenge in constructing owner-reported instruments that prove adequate measurement properties.

Despite the challenges to owners to estimate pain experienced by their dogs, psychometric testing of the CBPI has shown adequate construct and criterion validity to assess owner-perceived pain. Psychometric testing refers to the construction and evaluation of an instrument developed to assess an unobservable concept, such as chronic pain [9, 11] and health related quality of life [19] in dogs. During the development of the CBPI, an exploratory factor analysis (EFA) was used to explore the relationships among the observed variables (items in a questionnaire), and to study the construct and subdomains. Brown et al. [9] extracted two subdomains in the CBPI; pain severity and pain interference with function in daily activities, in a group of dogs with pain related to OA, prior to treatment with anti-inflammatory drugs [9]. Later, Walton et al. [12] repeated an EFA in another sample of dogs with pain related to OA. In their sample, all CBPI items loaded heavily on one component, which they did not define [12]. Psychometric testing of the CBPI has also shown good internal consistency [9], moderate to strong test–retest reliability and a clinically significant responsiveness to change in the owners’ perception of pain in dogs after anti-inflammatory treatment [9, 20, 21]. Nevertheless, when animal health-care professionals and researchers are selecting appropriate owner-reported outcome measures for pain associated to OA, considerations should be given to the purpose of the assessment and the context in which it is being used. The measurement properties, i.e. validity, reliability and responsiveness, should be established in the population of interest [4, 22, 23] and an instrument should be properly translated to the target language [24, 25]. The CBPI was originally validated in a group of untreated dogs with OA pain. To use the CBPI in a more diverse group of dogs with OA pain, e.g. dogs presented for physiotherapy, the instrument should to be psychometrically tested for its construct validity, and considered adequate. Construct validity concerns internal relationships, i.e. structural validity, and relationships to scores of other instruments or differences between known groups, i.e. hypothesis testing and cross-cultural validity [26]. The validity in a clinical target group, may differ from original psychometric testing. For instance, undergoing treatment, e.g. anti-inflammatory medication, may change the measurement properties of the CBPI, possibly threatening the validity of the CBPI as an outcome measure along the rehabilitation process. Dogs presented for physical rehabilitation in a veterinary primary care unit are a heterogeneous group of individuals and anti-inflammatory drugs are common treatment options in canine OA [1]. The psychometric properties of the CBPI has not yet been documented in such a diverse sample of dogs and our knowledge about whether the validity of the CBPI extends to a group including dogs that have ongoing anti-inflammatory treatment, i.e. are under pain management treatment, needs to be supplemented. In addition, the CBPI has not been translated from English to Swedish. Consequently, to assess the construct validity of the CBPI in a new target group, and to use the CBPI in the original target group, e.g. in a clinical protocol and for research, the CBPI questionnaire needs to be translated to the target language. In the present study, we hypothesized that the two-factor representation in the CBPI, pain severity and interference of pain with function, would be confirmed in a group of owners with dogs with OA pain presented for physical rehabilitation in a veterinary primary care unit. We also hypothesized that clinically sound dogs would show lower CBPI scores than OA dogs. Thus, the objectives of the present study were to translate the original CBPI and evaluate psychometric properties, in terms of internal consistency and construct validity, of the CBPI in a clinical sample of OA dogs referred for physiotherapy.

## Methods

### Study design

This study was an observational and cross-sectional study consisting of two groups of dogs that were consecutively recruited.

### Study group and procedure

To determine the size of the sample we used a subject-to-item ratio 5:1 [27, 28]. The subject-to-item ratio was determined by the number of CBPI items rated by the owners; hence 10 items generated a sample size of 50 dogs. The sample size was overestimated by 10% to cover possible losses and altogether we aimed for 60 dogs with OA. We aimed for approximately 20 dogs in a control group and when the number was reached, it became clear that the respondents in the control group scored mainly zero in pain severity and pain interference with function items, yielding no more information from the owner-perceived answers.

Sixty-one dogs referred from primary care veterinarians for physical rehabilitation interventions due to naturally occurring OA were included in the OA group. A group of 21 clinically sound dogs participated as controls. Of these, 19 clinically sound dogs and their owners also participated as controls in another research study conducted by two of the authors (PG and AE) (unpublished data). All dogs, in both groups, were clinically examined by a veterinarian prior to enrolment in the study. The OA dogs were diagnosed before they were recruited to the study. None of the control dogs had a history or current clinical evidence of OA. At a visit to a registered animal physiotherapist (AE), the clinical history was collected and the owners, whose dogs fulfilled the inclusion criteria, answered the Swedish version of the CBPI. The CBPI questionnaire was administered to the owners at the veterinary clinic and the owners were instructed according to the user guide available for the CBPI (www.canineBPI.com). Collection of the questionnaire was performed on the same occasion. Nineteen of the owners of the clinically sound dogs received the questionnaire from a veterinarian (PG). The inclusion criteria were as follows: dog >1 year of age, dog >9 kg body weight, clinical evidence of OA of at least one synovial joint, radiographic evidence of osteoarthritis of at least one synovial joint. The following were exclusion criteria: the owner completing the questionnaire lacked an understanding of written Swedish, other concurrent disease interfering with the dogs’ mobility, activity or health related quality of life. The dogs and the owners in the control group fulfilled the same inclusion and exclusion criteria as the OA group, except the clinical and radiographic evidence of OA. All dogs were client-owned and informed owner consent was obtained. The study protocols were approved by the Local Ethical Committee in Uppsala, Sweden (C81/12, C111/12, C17/2016).

### Instruments

Canine brief pain inventory is a 10-item questionnaire. The first four items consist of eleven-point (0–10) rating scales asking the owners to rate the pain intensity in their dogs during the last seven days, addressing pain “at its worst”, “at its least”, “on average” and “right now”. Zero indicates “no pain” and 10 represents “extreme pain”. The remaining six items cover the degree to which the owners rate the pain interference with function for their dog. In the interference items, 0 indicates “does not interfere” and 10 indicates “interferes completely”. CBPI scores are aggregated in two dimensions: (1) pain severity, using the four items (items 1–4) on pain intensity, and (2) pain interference, using the six items (items 5–10) on pain interference with function. The minimum sum of CBPI is 0 and the maximum sum in the pain severity items is 40 and in the pain interference 60. The sums of the two dimensions may be averaged to deliver a pain severity score and a pain interference score.

Body condition score was assessed by palpation and visual inspection by two of the investigators (AE and PG). A nine-point scale, reaching from one (severely underweight) to nine (obese), was used to assign the dogs to a body condition score, and in addition bodyweight was determined with digital scales [29]. Additional descriptive measures were collected on the same occasion that the owners answered the questionnaires (Table 1). The age (years) and body weight (kg) of the dogs were documented together with data on treatment with anti-inflammatory medication.

**Table 1:** Demographic data of the cohort (n = 79)

	OA group (n = 58)	Control group (n = 21)
Age (years)	7 (1–12)	5 (1–10)*
Body weight (kg)	30.4 (9–56)	26 (9–53)
Body condition score	5.5 (3–8)	4 (4–5)*
Anti-inflammatory medication	46 (79%)	0 (0%)
Gender (female)	25 (43%)	13 (62%)

Values are presented in median (range) and frequencies (proportions)

*OA* osteoarthritis

* Significant difference estimated with Mann–Whitney’s U test

### Translation and back translation

Permission to translate the CBPI into Swedish was obtained in a written consent from the copyright holder Dr. Dorothy Cimino Brown. Standard procedure for translation of instruments designed for self-reported outcome was used [25]. The English versions of the CBPI instrument were translated into Swedish with a forward and backward procedure as follows: a translation from English to Swedish was done from the original language, i.e. English, by two independent native Swedish persons who were fluent in the target language and who had good understanding of the original language. Further, the Swedish version of CBPI was back translated into the original language by two independent native English persons who were fluent in the original language and had good understanding of the target language. The translation processes described by Wild et al. [24] and Terwee et al. [25] were used as guidelines and the questionnaire was pretested in a pilot study on the target sample. The translations were reviewed until a consensus about a translated version was reached. The pilot study was not included in this study.

### Statistical analysis

Descriptive statistics comprised baseline data on age, bodyweight, body conditions score, on-going use of anti-inflammatory medication, presented as median (min–max) and frequencies (proportion). The pain severity sum, pain interference sum, total CBPI sum, pain severity score and pain interference with function score are presented as median (min–max). The internal consistency of the questionnaire was estimated to examine the extent to which items in the questionnaire correlated and measured the same concept. In accordance with Nunnelly and Bernstein [30] Cronbach’s α >0.70 was considered acceptable.

**Fig. 1:**
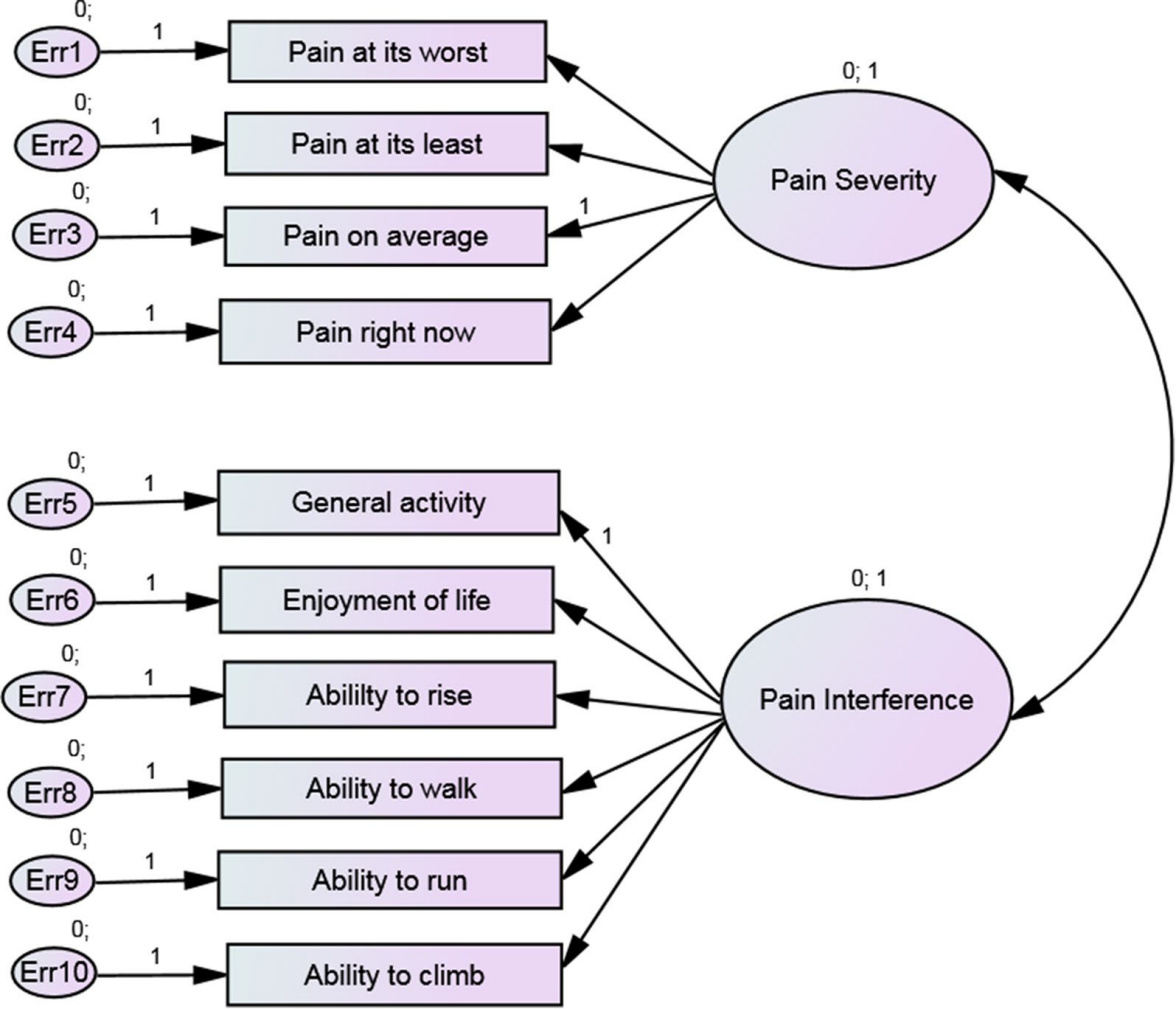
Confirmatory factor model for the two-factor solution

Construct validity (structural validity) was assessed by confirmatory factor analysis (CFA) and EFA [22, 25]. When there already is an accepted theory or empirical knowledge about the relationships between the latent, e.g. pain severity and pain interference, and observed variables, e.g. the items in the CBPI questionnaire, a confirmatory factor analysis (CFA) can be used to establish and possibly confirm construct validity [6, 7]. The CFA was used to test a specification of a model with several factors, based on previous research focusing on the factor structure by means of goodness-of-fit. A CFA by maximum likelihood method was conducted to test the hypothesis that the two-factor representation in the CBPI would be confirmed (Fig. 1).

The following goodness-of-fit indices were assessed: model-Chi^2^, degrees of freedom and Chi^2^/df, comparative fit index (CFI), root mean square error of approximation (RMSEA) with 90% confidence intervals, normed fit index (NFI), and parsimony adjusted normed fit index (PNFI). These fit indices show the extent to which the proposed model fit the data and thus indicate whether the specified structure may be confirmed. According to the guidelines by Hu and Bentler [31] acceptable model fit values are RMSEA values close to 0.06 or less; and CFI values are close to 0.90 or higher; NFI >0.95. The PNFI should be used in conjunction with NFI and could have values as low as 0.5 when NFI is as high as 0.9. Model Chi^2^ should lead to a high P value (*P* > 0.05) for a good model, and relative to its degrees of freedom, Chi^2^/df <2.0. The CBPI has a theoretical foundation that indicates two domains; pain intensity and pain interference with function [9], although Walton et al. [12] presented only one dimension. Based on the EFA presented by Walton et al. [12] and Brown et al. [9] we also tested a one-factor model with all CBPI items. Because the ordered data from CBPI items are not normally distributed and not on a quantitative measurement scale, we also estimated the models by bootstrapping and by Bayesian methods [32]. These methods do not change the goodness-of-fit indices, but do reveal differences in estimated factor loadings and variances.

We repeated an EFA by principal component model with subsequent varimax rotation, to study the interitem relationship and to explore the factor structure in our target group. Factors were extracted according to Kaiser’s rule; eigenvalues >1. Kaiser–Meyer–Olkin measure of sampling adequacy was calculated to assess whether our data was appropriate for factor analysis. Kaiser–Meyer–Olkin values between 0.5 and 0.7 was considered mediocre, 0.7–0.8 good, 0.8–0.9 high and above 0.9 excellent [33]. In addition, a significant Bartlett’s test of sphericity was also required for our data to be suitable for factor analysis [34].

When a marked proportion of the respondents score their dogs at the minimum, a floor effect occurs. Due to the notable item level and total sum floor effect in the group of OA dogs, we created a subgroup in which the owners reported pain related to OA, CBPI total sum ≥1. The CFA and EFA were repeated in the subgroup of dogs (n = 49).

By assessing for differences between sound dogs and dogs diagnosed with OA, using Mann–Whitney U test, the construct validity (hypothesis testing) and the ability of the CBPI to discriminate dogs with OA was tested [22, 25]. The nonparametric Mann–Whitney U test was used due to the non-normality of the ordered scaled data received from the CBPI items.

Values of *P* < 0.05 were considered statistically significant and two-tailed assessments were used for all analyses. SPSS (Version 20, IBM Statistical Package for Social Science Statistics for Windows, Armonk, NY: IBM Corp) was used for descriptive statistics group comparisons, internal consistency and EFA. AMOS (IBM, SPSS, AMOS 22.0., AMOS Development Corporation, Spring House, PA) was used for CFA.

## Results

Data from 79 adult dogs (38 females and 41 males) were included in this observational cross-sectional case–control study with the objective to translate the CBPI and validate the CBPI in a Swedish sample of OA dogs presented for physiotherapy. Inadequate completion of the CBPI questionnaire was present in three cases. Those were handled as internal missing values and the total completion rate was 97.5%. Translation of the CBPI showed that semantic equivalents were found in Swedish and that the conceptual meaning in the translated version of the questionnaire could be kept unchanged. There were significant demographic differences between control dogs and OA dogs in terms of age and body condition score. OA dogs were older and had higher body conditions scores. Most OA dogs (79%) had ongoing anti-inflammatory medication (Table 1). Descriptive information about the breeds included in the cohort can be found in Additional file 1.

### Internal consistency and floor effect

Cronbach’s α was 0.94 in the total CBPI, and 0.91 in the pain severity domain and 0.91 in the pain interference with function domain (n = 58). All items but “enjoyment of life” showed high levels of communality (>0.6) (Table 2). In the OA group (n = 58), more than 20% of the respondents rated floor value (score of 0) in all items, generating a floor effect. There was also a floor effect present in the pain severity sum and the pain interference with function sum (Table 2). In the group of owners rating presence of pain related to OA in their dogs (n = 49), the pain severity average (min–max) was 2.5 (0.0–5.8) and the pain interference with function average (min–max) was 2.3 (0.3–7.7). Including the owners that did not rate presence of pain related to OA (n = 58), the pain severity average (min–max) was 1.9 (0.0–5.8) and the pain interference with function average (min–max) was 2.1 (0.0–7.7).

**Table 2:** Pain severity (items 1–4) and pain interference with function (items 5–10) scores, percentage of minimum scoring and communalities for the CBPI items, pain severity sum, pain interference with function sum and the CBPI items totaled

All OA dogs (n = 58)				OA dogs with CBPI ≥1 (n = 49)			Control dogs (n = 21)		
CBPI item	Median (min–max)	% Floor^c^	Communalities	Median (min–max)	% Floor^c^	Communalities	Median (min–max)	% Floor^c^	Communalities
Pain at its worst^a^	3 (0–8)	24.6	0.76	4 (0–8)	12.2	0.74	0 (0–1)	95.2	NA
Pain at its least^a^	0 (0–5)	54.1	0.80	1 (0–5)	46.9	0.76	0 (0–0)	100	NA
Pain on average^a^	2 (0–6)	31.7	0.95	2 (0–6)	20.4	0.94	0 (0–0)	100	NA
Pain right now^a^	1 (0–6)	42.6	0.82	2 (0–6)	32.7	0.78	0 (0–0)	100	NA
General activity^b^	2 (0–10)	34.4	0.77	3 (0–10)	22.4	0.72	0 (0–0)	100	NA
Enjoyment of life^b^	1 (0–9)	44.1	0.57	1 (0–9)	34.7	0.53	0 (0–0)	100	NA
Ability to rise^b^	2 (0–9)	28.3	0.68	3 (0–9)	16.3	0.60	0 (0–0)	100	NA
Ability to walk^b^	1.5 (0–8)	31.7	0.84	2 (0–8)	20.4	0.82	0 (0–0)	100	NA
Ability to run^b^	2 (0–10)	31.7	0.81	2 (0–10)	20.4	0.78	0 (0–0)	100	NA
Ability to climb^2^	1 (0–10)	43.3	0.67	2 (0–10)	32.7	0.62	0 (0–0)	100	NA
PS sum	7.5 (0–23)	25.9	NA	10 (0–23)	12.2	NA	0 (0–1)	95.2	NA
PI sum	12.5 (0–46)	15.5	NA	14 (2–46)	0	NA	0 (0–0)	100	NA
CBPI total sum	19.5 (0–66)	15.5	NA	24 (2–66)	0	NA	0 (0–1)	95.2	NA

Values are presented as median (minimum–maximum). Proportion (%) of scoring minimum value (floor) value. Communalities showing proportions of variance for each item that can be explained by the two components, pain severity and pain interference with function, in the exploratory factor analysis by principal component analysis

*CBPI* canine brief pain inventory, *OA* osteoarthritis, *PS* pain severity (item 1–4), *PI* pain interference with function (item 5–10), *NA* not applicable

^a^Range 0–10 (no pain, extreme pain)

^b^Range 0–10 (does not interfere, interferes completely)

^c^% Scoring minimum value

### Construct validity; structural validity

In the CFA, both one- and two-factor models had similar CFI and RMSEA values. The CFI and NFI values were too low, the ratios Chi^2^/df were small and the RMSEA were too high to be acceptable in all models. Altogether, this indicates that the proposed models could not be confirmed based on our data and we thereby do not show any estimated factor loadings and covariances. The fit indices for the confirmatory factor models by maximum likelihood estimation method are displayed in Table 3. Analysis by bootstrap modelling and by Bayesian estimations led to estimates of factor loadings, variances and covariances. These estimates differed somewhat from the maximum likelihood estimates. These results are shown in Additional file 2.

**Table 3:** Fit indices for the confirmatory factor models by maximum likelihood estimation method in all dogs with pain associated with osteoarthritis (n = 58) and dogs with osteoarthritis pain and CBPI ≥1 (n = 49)

Model	Chi^2^	Df	Chi^2^/df	CFI	NFI	PNFI	RMSEA	90% CI
1-factor (all OA)	156.81	36	4.36	0.775	0.730	0.584	0.243	0.204–0.282
1-factor(OA CBPI ≥1)	123.66	36	3.44	0.769	0.708	0.567	0.225	0.183–0.269
2-factor (all OA)	162.97	36	4.53	0.763	0.720	0.576	0.249	0.211–0.288
2-factor (OA CBPI ≥1)	126.55	36	3.52	0.761	0.702	0.561	0.229	0.186–0.273

Fit indices estimated were Chi^2^

*df* degrees of freedom, *Chi* ^*2*^ */df* model *Chi* ^*2*^ related to its degrees of freedom, *CFI* comparative fit index, *NFI* normed fit index, *PNFI* parsimony adjusted normed fit index, *RMSEA* root mean squared error of approximation, *CBPI* Canine Brief Pain Inventory, *CI* confidence intervals, *OA* osteoarthritis

After a CFA we continued the analysis of the factor structure with an EFA (Table 4). Kaiser–Meyer–Olkin values of the CBPI were high, 0.88 in the whole group of dogs studied and 0.85 in the subgroup OA dogs with CBPI ≥1. Bartlett’s test of sphericity was significant, altogether indicating our data were suitable for EFA. Exploratory factor analysis by principal component analysis showed a one-component structure with an eigenvalue of 6.7, in the total OA group (n = 58). One component showed an eigenvalue of 0.99 and was extracted together with the first component. Those two components accounted for 76.8% of the total variance (66.9 and 9.9% respectively), suggesting an acceptable fit of a two-component structure. Interitem correlations were good (overall > 0.35) and mean interitem correlation was 0.79 for severity items and 0.60 for interference items. In the group of OA dogs with CBPI total sum ≥1 (n = 49) two components with eigenvalues >1 were extracted. These components accounted for 60.9 and 11.9% of the total variance of the CBPI respectively. Together the components accounted for 72.8% of the total variance. Mean interitem correlations was in total 0.55. For severity items mean interitem correlation was 0.75 and for interference items 0.53.

**Table 4:** Factor loadings in the exploratory factor analysis by principal component analysis with subsequent varimax rotation for the CBPI (two factors extracted) in all dogs with osteoarthritis (n = 58), and dogs with osteoarthritis and CBPI ≥1 (n = 49)

CBPI item	All OA dogs		OA dogs with CBPI ≥1	
	Factor 1	Factor 2	Factor 1	Factor 2
Pain at its worst	0.83	0.27	0.85	0.14
Pain at its least	0.87	0.22	0.85	0.21
Pain on average	0.90	0.38	0.91	0.34
Pain right now	0.82	0.38	0.80	0.37
General activity	0.66	0.58	0.66	0.53
Enjoyment of life	0.48	0.58	0.45	0.57
Ability to rise	0.59	0.57	0.57	0.52
Ability to walk	0.14	0.91	0.04	0.91
Ability to run	0.37	0.82	0.33	0.82
Ability to climb	0.45	0.69	0.42	0.66

*CBPI* canine brief pain inventory, *OA* osteoarthritis

### Construct validity; hypothesis testing

Clinically sound dogs differed from OA dogs by showing significantly lower CBPI total sum, and significantly lower pain severity and pain interference with function sums.

## Discussion

In this study, the CBPI was psychometrically tested in two groups of dogs to investigate internal consistency and construct validity in a Swedish context and in a heterogeneous group of dogs with pain associated with OA. The results from our study supplements the existing knowledge with the CBPI by confirming good to excellent internal consistency, the ability to discriminate OA dogs from clinically sound dogs and the number of components extracted in EFA. The number of components retained and the eigenvalues of each component were similar in the group of dogs with CBPI ≥1 and the group of dogs studied by Brown et al. [9] during the original development and psychometric testing of CBPI. The internal consistencies were also similar. In comparison, Cronbach’s α for the total CBPI sum was 0.91 in the present study and 0.92 in the study conducted by Brown et al. [9]. Cronbach’s α for severity of pain and pain interference with function were 0.91 and 0.91 respectively in our study, and 0.93 and 0.89 in the study by Brown et al. [9].

The demographic data showed statistical differences in age and body condition score between OA dogs and controls. Control dogs were slightly younger and had lower body condition score compared to OA dogs. In contrast to previous studies on the measurement properties of the CBPI the majority of dogs had ongoing treatment with anti-inflammatory medications, which is common in an applied clinical setting. Our more diverse study sample contributes to knowledge about the differences among individuals (Tables 1, 2). The median, minimum and maximum values of each CBPI item indicate that a considerable number of dogs are still reported with major OA symptoms, i.e. pain and functional impairments in: general activity, enjoyment of life, ability to rise, ability to run, and ability to climb. Our results are consistent with previous reports on the efficacy of anti-inflammatory drugs in dogs with OA, showing that pain control is not complete with one modality only [21, 35]. Although 79% of the OA dogs had ongoing on anti-inflammatory medication the canine owners still report presence of pain symptoms and that pain interferes with daily activities and mobility. The present study is not designed for the assessment of cross-cultural differences among dog owners in Sweden compared to dog owners in the US, since our sample is based on a new target group. However, the pain severity score, the pain interference with function score and the CBPI total sum (Table 2) in the present study allow for comparisons with the CBPI scores reported in several drug intervention studies for canine osteoarthritis [21, 35]. The pain severity score, pain interference score and the CBPI total sum in owners rating presence of pain related to OA in their dogs in our study are similar to those previously reported after two weeks of treatment with anti-inflammatory drugs [21, 35]. When the dog owners who did not rate presence of pain in their dogs were included, the CBPI scores and the present study were lower compared to the values reported to Brown et al. [21], but in the total CBPI sum was in consistence with the value reported by Wernham et al. [35]. The findings in the present study consist of a systematic examination of the CBPI in a new diverse target group, revealing an item level, as well as a total score, floor effect in dogs with pain related to OA. The proportion of respondents scoring zero in the pain severity sum was 12.2% in the group with CBPI ≥1. Floor effects of about 15–20% indicate that the true level of owner-perceived symptoms of pain would not be adequately represented by the CBPI in our sample [36]. Hence, there may be more variance in the concept, i.e. chronic pain, that may not be measured by the CBPI. It should be mentioned that the owner-reported ratings in the present study may be attributed to a response shift in the internal standards of the owners of the OA dogs undergoing pain management. And, as with the owners rating pain intensity in their dogs using a visual analogue scale [16], they may have a lack of recognition of subtle behavioural signs related to persistent OA pain in their dogs. The later may explain the higher proportion of floor effects in the pain severity items. Consequently, the changes in scores may be limited by the floor effect, and the responsiveness of the CBPI may be reduced because many dogs would not be able to change their item score despite the likelihood of clinical improvement. However, the sum of pain interference with six daily activities, i.e. general activity, enjoyment of life, rising to standing, walking, running, and climbing, showed no floor effect in the group of dogs rated CBPI >1 (Table 2), suggesting the pain interference items may be more sensitive to change in dogs undergoing multimodal OA management. However, it was not within the scope of this study to assess the responsiveness of the instrument.

The fit indices achieved in the CFA were not acceptable according to the guidelines by Hu and Bentler [31]. Because neither of our proposed CFA models could be confirmed, the hypothesis of the presented two-factor representation in the CBPI, was rejected as causal structure underlying the construct. The subsequent principal component analysis performed in our study showed that three of the CBPI items loaded equally on the two extracted factors, i.e. general activity, enjoyment of life and ability to rise, indicating that the items are influenced by both the pain severity and the pain interference factor. Since the factor loading is the correlation between the factor and the item, it seems like three of the pain interference items also may assess the pain severity factor in our sample. All the CBPI items are still important in the instrument because they show heavy positive loadings (>0.4), but the factor structure seems to differ from the original. In the original psychometric testing, Brown et al. [9] presented that item 1–4 in the CBPI questionnaire loaded heavy on pain severity, and item 5–10 loaded heavy on the pain interference factor. As indicated in Table 4 the factor loadings were rather equal in several items, and the one-factor and two-factor model analyzed in our CFA did not allow for the dual loading.

Since we performed a comprehensive linguistic translation process with high quality, according to guidelines for translation and cross-cultural adaption [24, 25], we do not relate the failure of the one-factor and the two-factor model in the CFA to the translation process. Instead we consider several other possible reasons why the original two-factor factor model in the CFA did not fit our Swedish sample. We know that our samples vary in comparison with the study that performed the original psychometric testing of CBPI because we also enrolled dogs with ongoing anti-inflammatory medication. Moreover, the proportion of the floor effect of the CBPI in our study group, in combination with a small sample size and equal factor loading in three of the interference items, may also have contributed to skewness in data leading to computational difficulties in the CFA. Analyzing ordinally scaled items as they were on a continuous scale may also have consequences for the results. The difference in estimates we found in the Bayesian estimation support this.

Our results highlight the importance of validated instruments to increase the certainty with which they accurately reflect what they are supposed to measure. The concepts within an instrument may differ with context, culture and/or time, which in turn may bias a study if the equality is not ensured across different target groups. The cohort included in this study consisted of canine owners who rated the severity of pain and the pain interference with function in dogs undergoing medical pain management, as well as dogs not undergoing medical treatment, which is highly applicable in clinical settings at veterinary primary care units and canine rehabilitation facilities. Indeed, it has been shown that ratings of the perceived level of pain in another individual is difficult and may differ in humans e.g. between parental caregivers and children experiencing pain [14]. Pain in dogs is hence not an easy phenomenon to quantify. The clinical picture of a dog with persistent OA pain or chronic pain behaviour associated with OA, includes changes in several dimensions and may be evaluated by deconstruction of the pain behaviour. In contrast to the CBPI, the Helsinki Chronic Pain Index is a chronic pain questionnaire that allows the canine owners to describe the behaviour of the dog rather than to rate the level of pain or the level of interference of pain on function [37, 38]. A description of each canine chronic pain behaviour under investigation may contribute to make the ratings more defined, which may overcome part of the subjectivity in rating a sensory and emotional pain experience of an animals [39, 40].

Our data showed a high level of communalities (all but one >0.6) in the EFA which we considered to be good enough to consistently reproduce the factor loadings even in a small sample. A limitation of our study was that although communalities were high in the EFA the sample size was small, but still fair, for factor analysis. However, according to the COSMIN checklist manual, our assessment of the structural validity of the CBPI in a diverse group of dogs with pain related to OA was excellent in all other aspects [22, 25].

## Conclusions

The quality of our comprehensive Swedish translation of the CBPI was high and the translated version is valid for use in the Swedish language. The findings of this study suggest satisfying psychometric properties in terms of high internal consistencies and ability to discriminate clinically sound dogs from OA dogs, of the CBPI in a heterogenous Swedish sample of dogs with pain related to OA. Although, animal health-care professionals should be aware of the potential floor effect in a sample similar to ours and that the interpretability of the results from the CBPI may be affected. As the pain interference with function items showed no floor effect in owners reporting pain ≥1 in their dogs, we suggest that part of the CBPI can be used separately. With regard to the EFA, three of the pain interference items, i.e. general activity, enjoyment of life and ability to rise, were also correlated with pain severity. Furthermore, based on the CFA the original factor structure in CBPI is not ideally suited to measure owner-perceived pain related to OA in our diverse sample of dogs, and the hypothesis of the presented two-factor structure was rejected. Further research needs to be conducted to determine whether the original psychometric results from CBPI can be replicated across different target groups and particularly with larger sample size.

## Additional files

**Additional file 1.** List of breeds included in the study cohort [osteoarthritis group (n = 58), control group (n = 21)] and the number of individuals in each breed.

**Additional file 2.** Estimates of factor loadings from confirmatory factor analysis models estimated by maximum likelihood method, bootstrap technique, and Bayesian method.
