# Erratum to: Psychometric evaluation of the canine brief pain inventory in a Swedish sample of dogs with pain related to osteoarthritis

**DOI:** 10.1186/s13028-017-0319-7

**Published:** 2017-07-25

**Authors:** Ann Essner, Lena Zetterberg, Karin Hellström, Pia Gustås, Hans Högberg, Rita Sjöström

**Affiliations:** 1 Department of Neuroscience, Faculty of Physiotherapy, Uppsala University, Husargatan, Biomedical Centre, Box 593, 751 24 Uppsala, Sweden; 2 Evidensia Djurkliniken Gefle, Norra Gatan 1, 803 21 Gävle, Sweden; 3 Department of Clinical Sciences, Faculty of Veterinary Medicine and Animal Husbandry, Swedish University of Agricultural Sciences, Box 7054, 750 07 Uppsala, Sweden; 4 Department of Health and Caring Sciences, Faculty of Health and Occupational Studies, University of Gävle, 801 76 Gävle, Sweden; 5 Unit of Research Education and Development, Region Jämtland Härjedalen, Box 654, 831 27 Östersund, Sweden; 6 Department of Community Medicine and Rehabilitation, Faculty of Physiotherapy, Umeå University, 901 87 Umeå, Sweden

## Erratum to: Acta Vet Scand (2017) 59:44 DOI 10.1186/s13028-017-0311-2

After publication of this paper [1] it was noticed that there was an error in the corresponding author’s surname which was duplicated. The corresponding author’s name should read: Ann Essner.

The original article was corrected.

The publisher apologises for this error.
