# Supplementary material for: Psychometric evaluation of the canine brief pain inventory in a Swedish sample of dogs with pain related to osteoarthritis
Source: Acta Vet Scand. 2017 Jul 1;59:44. doi: 10.1186/s13028-017-0311-2 (PMC5493851; doi:10.1186/s13028-017-0311-2)
Supplement: Supplementary file 1 — Additional file 1. List of breeds included in the study cohort [osteoarthritis group (n = 58), control group (n = 21)] and the number of individuals in each breed. [file 13028_2017_311_MOESM1_ESM.docx]

**Additional file 1** List of breeds included in the study cohort (osteoarthritis group (n=58), control group (n=21)) and the number of individuals in each breed.

| **OA-group (n=58)** | | |
| --- | --- | --- |
| **Breed** | **Number** | **Proportion (%)** |
| American Staffordshire Terrier | 1 | 1.7 |
| Border Collie | 1 | 1.7 |
| Boxer | 2 | 3.4 |
| Cavalier King Charles Spaniel | 1 | 1.7 |
| Dobermann | 1 | 1.7 |
| English Springer Spaniel | 3 | 5.2 |
| Flat Coated Retriever | 3 | 5.2 |
| French Bulldog | 1 | 1.7 |
| German Pointer | 1 | 1.7 |
| German Shepherd Dog | 5 | 8.6 |
| Golden Retriever | 9 | 15.4 |
| Irish Red Setter | 1 | 1.7 |
| Labrador Retriever | 10 | 17.2 |
| Lagotto Romagnolo | 2 | 3.4 |
| Leonberger | 1 | 1.7 |
| Mixed-breed | 7 | 12.1 |
| Nova Scotia Duck Tolling Retriver | 1 | 1.7 |
| Rottweiler | 2 | 3.4 |
| Shetland Sheepdog | 2 | 3.4 |
| Staffordshire Bull Terrier | 4 | 6.9 |

| **Control group (n=21)** | | |
| --- | --- | --- |
| **Breed** | **Number** | **Proportion (%)** |
| Australian Shepherd | 2 | 9.5 |
| Bearded Collie | 1 | 4.8 |
| Belgian Shepherd Dog | 1 | 4.8 |
| Bernese Mountain Dog | 1 | 4.8 |
| Border Collie | 3 | 14.3 |
| Border Terrier | 1 | 4.8 |
| Boxer | 2 | 9.5 |
| Flat Coated Retriever | 1 | 4.8 |
| Golden Retriever | 2 | 9.5 |
| Great Dane | 1 | 4.8 |
| Labrador Retriever | 3 | 14.3 |
| Mixed-breed | 2 | 9.5 |
| Rottweiler | 1 | 4.8 |
