# Supplementary material for: Psychometric evaluation of the canine brief pain inventory in a Swedish sample of dogs with pain related to osteoarthritis
Source: Acta Vet Scand. 2017 Jul 1;59:44. doi: 10.1186/s13028-017-0311-2 (PMC5493851; doi:10.1186/s13028-017-0311-2)
Supplement: Supplementary file 2 — Additional file 2. Estimates of factor loadings from confirmatory factor analysis models estimated by maximum likelihood method, bootstrap technique, and Bayesian method. [file 13028_2017_311_MOESM2_ESM.docx]

**Additional file 2** Estimates of factor loadings from confirmatory factor analysis models estimated by maximum likelihood method, bootstrap technique, and Bayesian method.

|  | **All OA dogs (n=58)** | | | | | | **OA dogs with CBPI ≥1 (n=49)** | | | | | |
| --- | --- | --- | --- | --- | --- | --- | --- | --- | --- | --- | --- | --- |
|  | **1-factor** | | | **2-factor** | | | **1-factor** | | | **2-factor** | | |
| **CBPI item** | **ML** | **Bootstrap** | **Bayes** | **ML** | **Bootstrap** | **Bayes** | **ML** | **Bootstrap** | **Bayes** | **ML** | **Bootstrap** | **Bayes** |
| Pain at its worst | 1.00 | 1.00 | 1.00 | 1.43 | 1.42 | 1.51 | 1.00 | 1.00 | 1.00 | 1.28 | 1.26 | 1.37 |
| Pain at its least | 1.11 | 1.08 | 1.22 | 0.88 | 0.87 | 0.95 | 1.18 | 1.14 | 1.32 | 0.96 | 0.94 | 1.04 |
| Pain on average | 1.60 | 1.57 | 1.76 | 1.00 | 1.00 | 1.00 | 1.59 | 1.56 | 1.78 | 1.00 | 1.00 | 1.00 |
| Pain right now | 1.38 | 1.37 | 1.53 | 1.09 | 1.07 | 1.17 | 1.40 | 1.38 | 1.58 | 1.14 | 1.11 | 1.24 |
| General activity | 2.02 | 2.02 | 2.22 | 1.00 | 1.00 | 1.00 | 1.93 | 1.93 | 2.17 | 1.00 | 1.00 | 1.00 |
| Enjoyment of life | 1.38 | 1.38 | 1.53 | 1.19 | 1.17 | 1.28 | 1.35 | 1.36 | 1.53 | 1.24 | 1.20 | 1.35 |
| Ability to rise | 1.84 | 1.84 | 2.04 | 1.61 | 1.58 | 1.73 | 1.65 | 1.65 | 1.85 | 1.54 | 1.49 | 1.66 |
| Ability to walk | 1.09 | 1.10 | 1.21 | 1.12 | 1.10 | 1.22 | 0.87 | 0.89 | 1.00 | 1.08 | 1.03 | 1.18 |
| Ability to run | 1.82 | 1.83 | 2.02 | 1.74 | 1.71 | 1.87 | 1.68 | 1.70 | 1.90 | 1.79 | 1.74 | 1.95 |
| Ability to climb | 1.57 | 1.57 | 1.75 | 1.49 | 1.46 | 1.61 | 1.44 | 1.45 | 1.63 | 1.53 | 1.46 | 1.66 |
| CBPI, canine brief pain inventory; ML, maximum likelihood; OA, osteoarthritis; Bayes, Bayesian method. | | | | | | | | | | | | |
